# Global Monkeypox Virus Outbreak 2022: A Bibliometric Analysis

**DOI:** 10.7759/cureus.37107

**Published:** 2023-04-04

**Authors:** Syeda Tayyaba Rehan, Hassan Ul Hussain, Kanwal Ashok Kumar, Mahnoor Sukaina, Zayeema Khan, Abdulqadir J Nashwan

**Affiliations:** 1 Department of Internal Medicine, Dow University of Health Sciences, Civil Hospital Karachi, Karachi, PAK; 2 Department of Medicine, Dow University of Health Sciences, Karachi, PAK; 3 Department of Medicine, Karachi Medical and Dental College, Karachi, PAK; 4 Department of Internal Medicine, Dow University of Health Sciences, Karachi, PAK; 5 Department of Nursing, Hamad Medical Corporation, Doha, QAT

**Keywords:** bibliometric analysis, zoonotic infectious disease, orthopoxvirus family, mpx, monkeypox virus

## Abstract

Monkeypox is a rare zoonotic disease caused by the monkeypox virus, which spreads by direct contact mainly, thus having the propensity to cause future epidemics. The current review aimed to provide an up-to-date literature analysis for evaluating scientific data on monkeypox. A bibliometric analysis was conducted through eight electronic databases. The search period was from May 2022 to December 2023. All the articles were exported to Mendeley (Elsevier, Amsterdam, Netherlands). The literature search resulted in 415 relevant research articles. The growth of publications gradually rose, initiated in January 2022, leading to a rapid upsurge in May 2022. A total of 409 documents reported the number of citations, with two articles documenting the highest number, ranging from 146-150 and 216-220. The European region (EURO) dominated in publishing research articles on monkeypox, with the United States having the highest number of reports (n = 41; 9.87%), followed by the United Kingdom (n = 35; 8.43%) and Italy (n = 15; 3.61%). There were 82 funding agencies that funded 44 research articles, whereas 371 were not funded by any funding agency. Our analysis has presented the outline of the research articles published on monkeypox virus-related literature during the current outbreak. Research articles should be financially and administratively supported. Future research is required to expand research on the monkeypox virus, as there is a growing demand for original articles.

## Introduction and background

Monkeypox is a rare zoonotic disease caused by a virus that belongs to the *Orthopoxvirus* genus in the family Poxviridae. This virus has two genetic variants: one in Central Africa (more severe and with higher mortality) and one in West Africa [1]. In 2018, a few travel-associated cases of human-to-human transfer emerged in the United Kingdom when a healthcare worker got infected by a patient [2]. The latest monkeypox outbreak in the United Kingdom began with an individual who traveled from Nigeria in May 2021 and developed a rash on the face [3]. Skin samples tested by polymerase chain reaction (PCR) showed infectivity with the monkeypox virus [3].

Furthermore, in May 2021, a family member residing with a patient developed similar symptoms and was also confirmed to be infected with the monkeypox virus [3]. The World Health Organization (WHO) predicted a dramatic increase in cases in previously non-endemic areas with the emerging trend of viral spread in 2021. As a result, approximately 105 member states covering six WHO regions have been robustly affected by the monkeypox virus since January 2022. However, the weekly report by WHO suggests an 18.7% decline in cases in September [4].

The virus poses a serious issue because, just like COVID-19, monkeypox disease spreads by direct contact, thus having the propensity to cause future epidemics. Monkeypox virus is transmitted mainly through direct (human to animal or human to human) communication. On the other hand, droplet transmission necessitates prolonged face-to-face contact and is also transmitted to newborns via the placental route [1]. The vital route is the transmission by aerosol, with the additional risk of spreading via contact with the infected individuals' lesion exudate or body fluids [5]. In 2019, the WHO reported a newly developed vaccine that got approval to prevent monkeypox infection. Imvamune, a vaccine for smallpox, has been permitted for monkeypox as both viruses are closely related. However, it has been beyond the reach of the general population [1]. Standard treatment for monkeypox infection is not available yet. Vaccines play a vital role in preventing the risk of monkeypox infections [6]. Cidofovir, or vaccine immunoglobulin (VIG), is efficacious in controlling the transmission. Furthermore, ST-246 and smallpox vaccines also benefit patients [6]. Moreover, a newly developed vaccine (modified vaccinia Ankara-Bavarian Nordic {MVA-BN}) and a monkeypox treatment with tecovirimat were approved in 2019 and 2022 [1]. However, global nonavailability may cause a burden of disease.

Bibliometric analysis tends to summarize large data and detect where lies the paucity of information and research gap. Several analyses have been carried out on different outbreaks and proved to be of great interest in tracking the literature gap [7-9]. We intend to present an updated bibliometric analysis of articles published on the monkeypox virus and its preceding outbreak from 2021 to 2022 to minimize the literature gap and as a call to action for researchers, respective regions, and authorities. This paper aims to evaluate the scientific data and published discoveries on the monkeypox virus. Furthermore, we aim to deduce patterns of publications and the months of the highest publications on the monkeypox outbreak.

## Review

Methodology

Eight electronic databases (Medline via PubMed, Scopus, ScienceDirect, Cochrane Library, Web of Science, Google Scholar, Education Resources Information Center {ERIC}, and Ovid) were used to recruit relevant articles for the current bibliometric analysis. Seven databases (Medline via PubMed, Scopus, ScienceDirect, Cochrane Library, Web of Science, ERIC, and Ovid) allowed both basic and advanced searching options, while one of them (Google Scholar) allowed only basic searching.

Literature Search and Search Strategy

Five databases were searched from May 2022, when the Centers for Disease Control and Prevention (CDC) reported the first monkeypox virus case of the current outbreak, to December 2022, while on the remaining three search engines (PubMed, ScienceDirect, and Ovid), the search was expanded until December 2023 as per the availability of databases' advanced searching features, without placing any language or regional restrictions. For the literature search, we used the following Medical Subject Heading (MeSH) terms: "monkeypox," "monkeypox virus," "MPV," "MPXV," and "hmpv." PubMed, ScienceDirect, and ERIC used similar search strategies, as did Cochrane Library, Web of Science, and Scopus. However, a unique strategy was used for Ovid. The search query was formed mainly based on a title search with the goal of selecting only articles directly related to the current wave of the monkeypox virus.

Bibliometric Indicators

**Figure 1:**
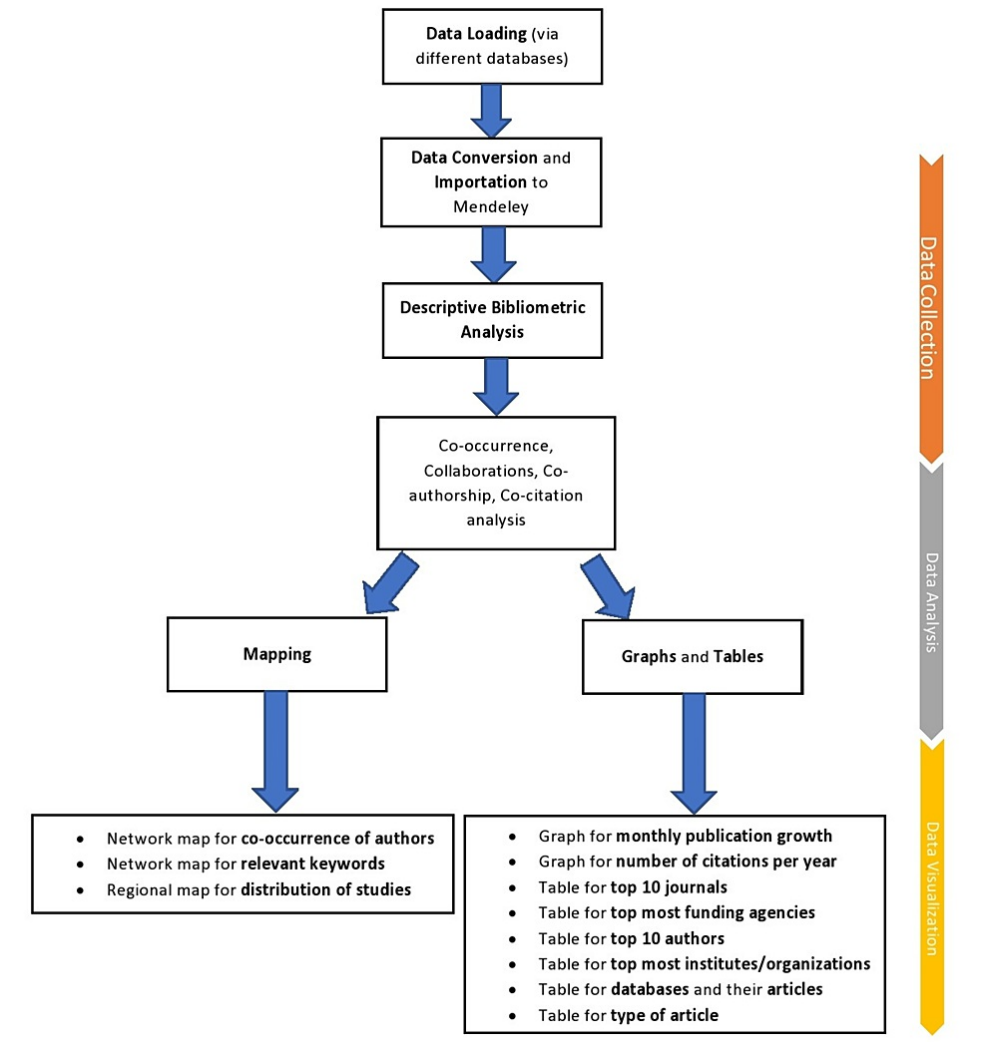
Steps for bibliometric analysis

All the articles were retrieved as RIS file types and then exported to Mendeley Desktop (version 1.19.8) (Elsevier, Amsterdam, Netherlands) [10], where the duplicates were searched for and removed on detection. The relevant studies were then independently evaluated by two reviewers (HUH and STR), first based on the title and abstract, followed by a full-text assessment to confirm their relevance. Group discussions were carried out to resolve discrepancies, if any. The concordance rate between the reviewers was 95%. All the irrelevant studies (without MeSH terms) were excluded from our bibliometric analysis. Data from the finalized articles was then exported to Microsoft Excel (Microsoft® Corp., Redmond, WA). The data included the type of article, first author's name, month and year of publication, journal name, country of publication, WHO region, number of citations, institutions/organizations, mode of access (open or closed), and funding agencies. Our bibliometric analysis carried out three vital steps (Figure 1).

The retrieved literature was also exported to the VOSviewer program (Leiden University, Leiden, Netherlands) to create network visualization maps [11]. The strength of the co-authored article was presented as "total link strength" (TLS), which is automatically given by VOSviewer upon mapping the research activities of selected authors. The TLS is proportional to the extent of co-authored documents and occurring keywords, where a higher TLS value indicates a greater co-authored document or relevance of keywords. To evaluate the growth of publications in the current outbreak of 2022 while operating the data extraction, we reviewed the journal's website to extract the month of the publication. A detailed bar chart was designed based on the frequency of documents endorsed by various institutes and organizations to look for the most active institutes and organizations. The most active funding agencies were based on the support and funds granted to researchers to conduct research related to the monkeypox virus.

Distribution Based on Countries and WHO Regions

For the geographical distribution of the included studies, the WHO regional classification was used: the region of the Americas (AMRO), the European region (EURO), the Western Pacific region (WPRO), the Eastern Mediterranean region (EMRO), the South-Eastern Asia region (SEARO), and the African region (AFRO) [12]. The finalized 415 articles in the analysis were manually screened for regional classification according to the WHO regions for territory and country extraction. The map for geographical distribution was designed using an online map designing software (https://www.mapchart.net) [13]. The world map indicated the countries that reported the monkeypox virus publications. The map key details the distribution on the map.

The Number of Citations of the Included Documents

We evaluated the number of citations of the included articles and summarized them in a graph. We initiated the data extraction of included articles and their citations that were exported from Google Scholar, PubMed, ResearchGate, and journal websites. We ranked the range with citation numbers from zero to 250.

**Figure 2:**
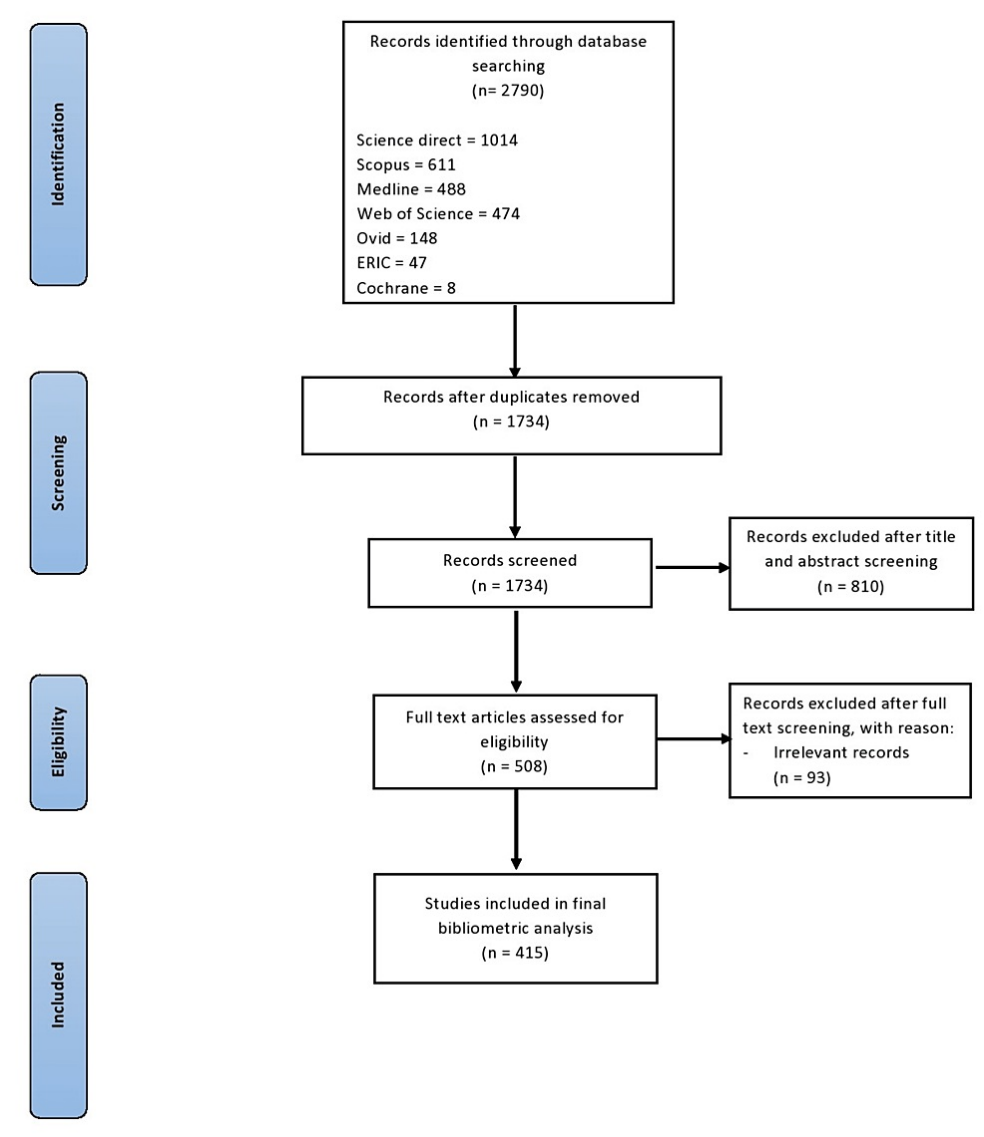
Summary of literature search ERIC: Education Resources Information Center

The initial search of all the databases yielded 2790 studies, out of which 1790 articles were removed after duplicate checking. Further, 810 articles were excluded after the title and abstract screening. After a full-text review of 508 studies, 415 were found relevant in our bibliometric analysis. A summary of the literature search is presented in Figure 2. Out of 415 articles, 348 were open access.

Table 1 shows the number and percentages of articles recruited from each database during the initial literature search. Among the total 415 articles, 22 types of articles were identified in which the letter to the editor (n = 69; 16.62%) had the highest frequency as compared to other study designs.

**Table 1:** Included documents and their relevant databases ERIC: Education Resources Information Center

Databases	Number of articles	% (n = 2790)
Medline via PubMed	488	17.49
ScienceDirect	1014	36.34
ERIC	47	1.68
Ovid	148	5.30
Cochrane	8	0.28
Web of Science	474	16.98
Scopus	611	21.89

Table 2 presents a comprehensive summary of the study designs of the included studies.

**Table 2:** Summary of study design for the recruited articles ^1^Miscellaneous represents viewpoint, clinical image, short article, web exclusive, untranslated articles, health agency update, test analytic study, satire, enhanced surveillance study, press, note, survey, resident forum, flash information, dispatch, report, feature article, correction article, and important updates and developments

Type of document	Frequency	% (n = 415)
Letter to the editor	69	16.62
Miscellaneous^1^	45	10.84
Editorial	42	10.12
News	41	9.87
Comment	36	8.67
Correspondence	35	8.43
Review	31	7.46
Case report	30	7.22
Short communication	23	5.54
Observational study	18	4.33
Opinion	10	2.40
Perspective	09	2.16
Book	04	0.96
Bibliometric analysis	03	0.72
In brief	03	0.72
Epidemiological study	02	0.48
Phylogenomic study	02	0.48
Experimental study	02	0.48
Meta-analysis	01	0.24
Systemic review	01	0.24
Qualitative study	01	0.24
Case-only study	01	0.24

Growth of Publications

**Figure 3:**
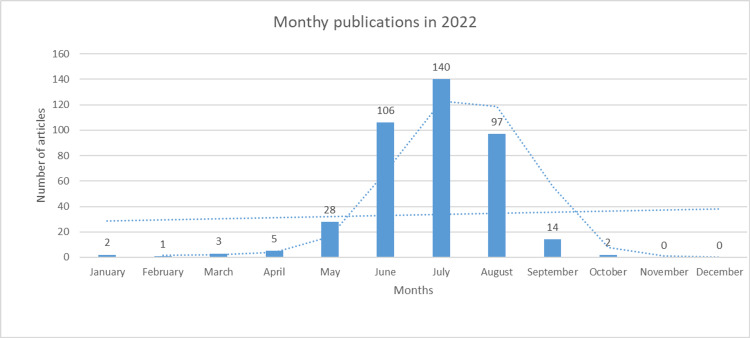
Monthly publication bar chart depicting the number of articles published in each month in 2022

The first case of monkeypox virus was reported in the 2021 outbreak in the United Kingdom from an individual with a travel history from Nigeria [14]. Until September 14, 2022, a robust surge in daily cases is documented by the WHO to be 59147 laboratory-confirmed cases and mortality in 22 individuals globally [4]. The alarming exponential rise in monkeypox transmission in 2022 intrigued research scientists and made the community aware of the deadly virus. From the results of our bibliometric analysis, the gradual rise in publication rate on the monkeypox virus was initiated in January 2022, with a rapid upsurge in the number of publications in May 2022. As the WHO remarked, the incidence of the outbreak in non-endemic regions such as Europe and North America started in May 2022 [15]. There was a marked incline in the number of publications in June, which is n = 106 (26.6%), of that year, as a sudden notable inclination is revealed in Figure 3, while the highest number of publications was reported in July 2022 (n = 140 {35.1%}). As elucidated in Figure 3, a dramatic increase in the bar and trend line is at its peak for July. And a slow decline in publications is in August 2022 (n = 97 {24.3%}), and progressively fewer publications are in September 2022 (n = 14 {3.5%}), and only 2 (0%) is available in October 2022.

Active Journals

Among the 118 journals in our analysis, Travel Medicine and Infectious Disease ranked first. This was the leading journal with the highest number of documents (n = 21), followed by BMJ (n = 19) and Annals of Medicine and Surgery (n = 16). Detailed information on the top 10 active journals is listed in Table 3.

**Table 3:** The number of articles published by the top 10 active journals and the journals' affiliated countries

Rank	Journals	Frequency	% (n = 415)	Country affiliation
1	Travel Medicine and Infectious Disease	21	5.06	United States
2	BMJ	19	4.57	United Kingdom
3	Annals of Medicine and Surgery	16	3.85	Netherlands
4	The Lancet	14	3.37	United Kingdom
5	International Journal of Surgery	14	3.37	Netherlands
6	The Lancet Infectious Diseases	12	2.89	United Kingdom
7	Nature	11	2.65	United Kingdom
8	Science	11	2.65	United States
9	Journal of Medical Virology	10	2.40	United States
10	Journal of Infection	8	1.92	United Kingdom

Active Authors

A total of 336 first authors participated in publishing various types of documents on monkeypox. The three top active authors were from Kazakhstan, Germany, and Thailand. Max Kozlov was the first-ranked active author, publishing seven studies, followed by Kai Kupferschmidt (n = 6) and Eujittika Mungmunpuntipantip (n = 6). A total of 70 research articles were single-authored.

Active Institutes/Organizations

There were 44 (10.60%) studies given support from different institutes, whereas 371 (89.39%) articles had no support from the institutions. Among 64 institutions, the Centers for Disease Control and Prevention was the most active institute, which provided support to eight research articles, followed by the Department of Infectious Disease (n = 3) and the National Institute for Infectious Diseases "Lazzaro Spallanzani" (an institute for hospitalization and treatment of a scientific nature {IRCCS}) (n = 2).

Active Funding Agencies

There were 82 funding agencies in the recruited studies that funded 44 (10.60%) articles, whereas 371 (89.39%) articles were not financed by any funding agency. Among the funded articles, the National Key R&D Program of China ranked the highest funding body and granted funds to four of the included articles.

Co-author and Keyword Co-occurrence

**Figure 4:**
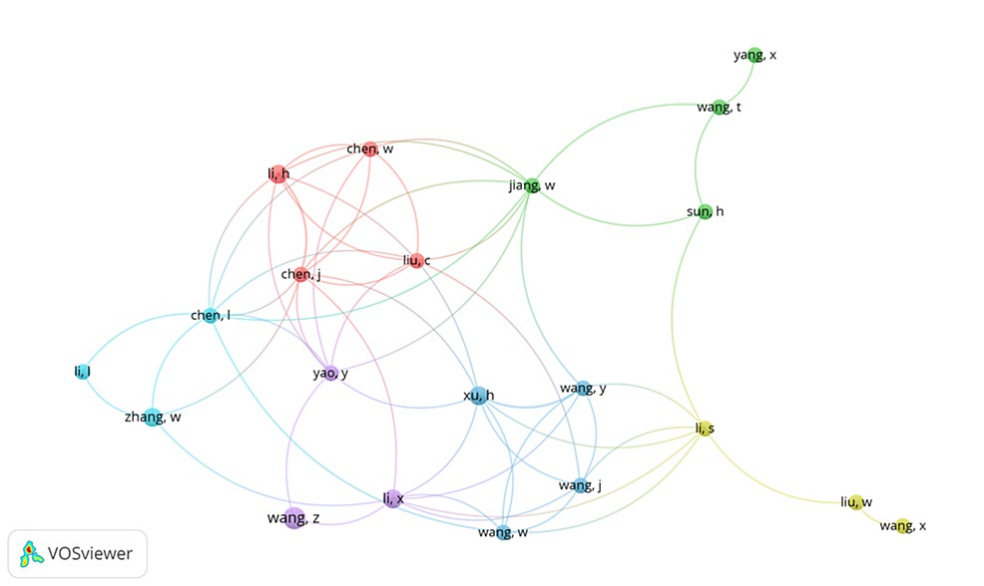
Co-author network visualization map with 21 items, a total link strength of 59, and six clusters Nodes with similar colors represent clusters of similar themes Generated via VOSviewer (https://www.vosviewer.com/)

**Figure 5:**
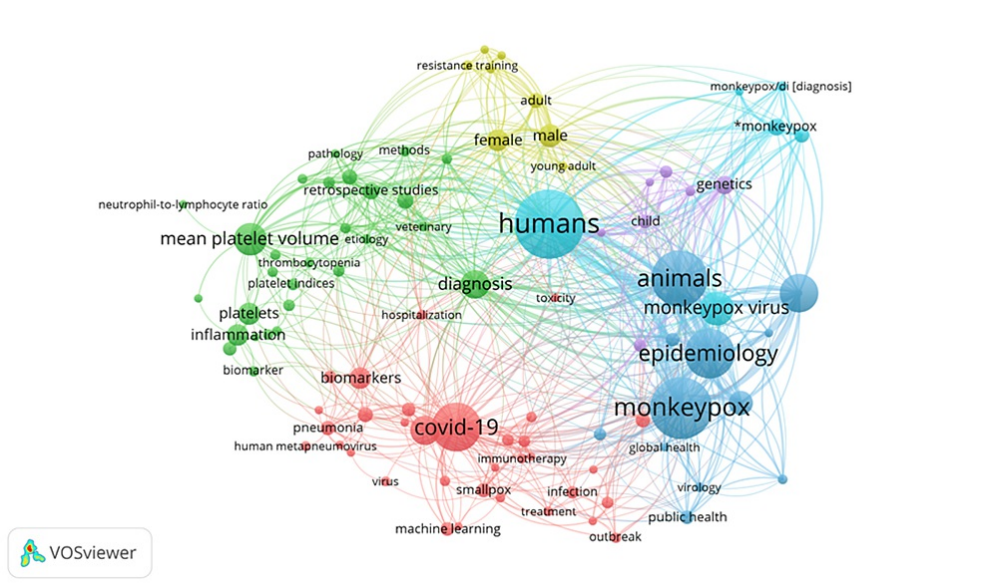
Network visualization map of keyword co-occurrence showing 94 items, six clusters, and a total link strength of 2552 Nodes with similar colors represent clusters of similar themes Generated via VOSviewer (https://www.vosviewer.com/)

The number of documents authored for the co-author map was set to three as a recruiting limit. Out of the 9181 authors, 108 authors met the threshold. Further, the VOSviewer generated a co-author map for 21 items with a link strength of 59 and six clusters. For the keyword co-occurrence map, the minimum number of keyword occurrences was set as five. Out of 3866 keywords that met the threshold, 126 were recruited via this criterion. A VOSviewer keyword co-occurrence map was generated, showing 94 items with six clusters and a total link strength of 2552. Figures 4-5 represent the co-author and keyword co-occurrence maps, respectively.

Distribution Based on the WHO Regions and Active Countries

**Figure 6:**
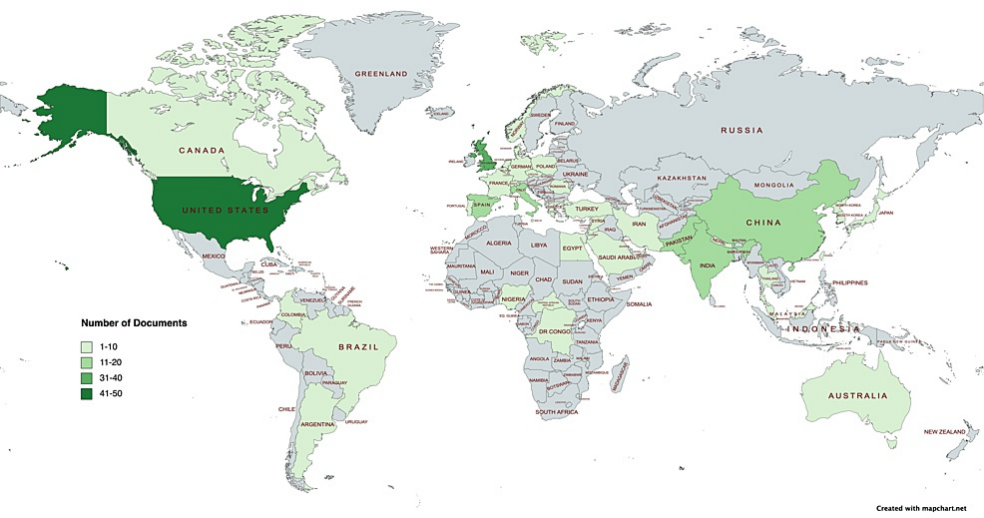
Geographical distribution of documents based on countries The colored areas on the world map represent countries that have published research on the monkeypox virus. The map key indicates the number of articles according to the color. The class (21-30) had no eligible articles; therefore, it is not displayed on the map

The detailed analysis of the included articles indicated that the European region had the highest contribution to the existing literature on the monkeypox virus (n = 95; 22.89%). The African region had the least contribution (n = 8; 1.92%). The detailed regional division of documents is presented in Figure 6.

Table 4 shows the top 10 active countries publishing documents in monkeypox virus-related literature. The list is dominated by countries from EURO, but the United States published the maximum number of articles (n = 41; 9.87%). The United States has the highest percentage of documents, followed by the United Kingdom (n = 35; 8.43%) and Italy (n = 15; 3.61%).

**Table 4:** Top 10 active countries in publishing monkeypox-related articles

Serial number	Active countries	Number of documents	% (N = 415)
1	United States	41	9.87
2	United Kingdom	35	8.43
3	Italy	15	3.61
4	Spain	13	3.13
5	China	13	3.13
6	India	11	2.65
7	Pakistan	11	2.65
8	Iran	07	1.68
9	Germany	07	1.68
10	Nigeria	04	0.96
11	France	04	0.96
12	Canada	04	0.96
13	Egypt	04	0.96
14	Portugal	04	0.96
15	Colombia	04	0.96
16	Netherlands	04	0.96

The Number of Citations

**Figure 7:**
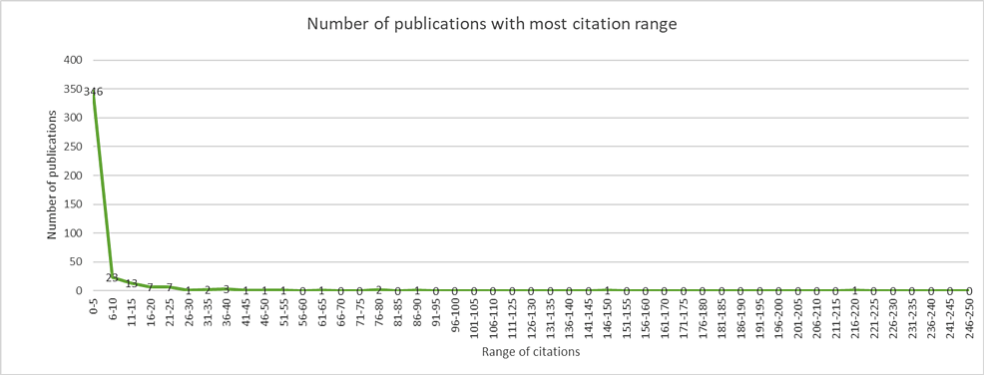
The x-axis demonstrates the ranges of citations, starting from 0-5 to 246-250. The y-axis signifies the number of publications starting from January 2022 and continuing to December 2022

We found that 411 studies reported the number of citations from the final included articles, of which most articles reported citations ranging from zero to five, which is 346 (84.5%), followed by 23 (5.5%) articles with citation ranging from six to 10 and 13 (3.3%) articles ranging from 11 to 15 citations. Furthermore, citation ranges of 16-20 and 21-25 are reported in seven (1.5%) articles, three (0.7%) for ranges of 36-40, and two (0.5%) for ranges of 31-35. Moreover, one (0.2%) was documented for citation ranges of 26-30, 41-45, 51-60, 61-65, and 86-90, including the two highest numbers of citations ranging from 146 to 150 and 216 to 220. From the graph (Figure 7), an exponential incline in the number of publications deduces that the highest rate of citations encompasses between zero and five citations. A sudden decline is notable as the number of publications decreases by 78.8% as the number of citations increases on the x-axis.

Bibliometric discussion

This bibliometric analysis was carried out to analyze and provide an overview of the current published literature on the monkeypox virus. The monkeypox outbreak is the first multination outbreak in non-endemic countries, first identified in May 2022 [16]. This makes this research critical for identifying the current gaps in the literature on the monkeypox virus. Our bibliometric research looked at papers from eight different databases. ScienceDirect produced the most papers (1014), with 348 of the 415 evaluated articles being open access. Most of the papers about the monkeypox virus were published in Travel Medicine and Infectious Disease. This journal published 21 of the 415 articles included. According to our findings, editorial articles are the most common form of an article on the monkeypox virus. This bibliometric analysis revealed a scarcity of original studies and surveys on the monkeypox virus.

A previous bibliometric assessment of the current literature on the monkeypox virus was published in May 2022 [17]. In contrast, the current analysis includes many databases using advanced search options, making it the most recent bibliometric analysis of the rapidly spreading monkeypox disease.

Furthermore, we extended our search through December 2023, as opposed to Lin et al.'s bibliometric analysis, which was conducted until June 2022 [18]. This bibliometric evaluation examined the top 10 most active countries based on the WHO geographical distribution, identifying the most active regions. Despite the WHO reporting 404 cases and seven deaths between January and August 2022, the African WHO region was the least productive regarding monkeypox virus-related literature. Similarly, the region of the Americas did not make sufficient progress regarding monkeypox-related papers, with 20438 cases and two deaths recorded [19]. Regional authors and funding institutions in Africa and the Americas may work together to advance the monkeypox virus research agenda. Our bibliometric study adds to Rodriguez-Morales et al.'s bibliometric analysis by including one-of-a-kind cluster map that highlights major author and keyword occurrences in the published literature providing a snapshot of the most researched avenues related to the monkeypox virus [17].

Finally, the COVID-19 pandemic has highlighted the importance of taking swift action to control the spread of diseases before they become widespread [20]. The current surge in monkeypox cases emphasizes the need for preparedness and caution in the early stages of an outbreak. Proper countermeasures implemented early on can improve patient outcomes, result to less-crowded hospitals, and prevent the worst-case scenario [21,22].

Future Implications

The current study investigated and evaluated global productivity in monkeypox virus research. To better understand the monkeypox outbreak in non-endemic countries, we discovered a critical shortage of original studies and investigations financed by health institutions and organizations. Funding agencies should be proactive in offering money for large-scale research on the monkeypox virus. The existing literature is dominated by editorial and commentary articles, which could be attributed to a lack of initiative from funding agencies to fund original research. The African WHO region contributed the least to the existing literature. Regional authors and organizations should pay closer attention to the research findings of top overseas journals and institutions to propose fresh avenues for regional monkeypox virus study. The monkeypox virus outbreak is causing a global healthcare problem, and additional efforts should be made to expand the existing material. Journals and funding bodies that do not contribute to the publication of large-scale research on monkeypox viruses should take prompt action [23,24].

Strengths and Limitations

To exclude irrelevant publications from our analysis, all contributing authors checked the title and abstract of the included studies. This has significantly decreased the number of false results from our analysis. Our review covered articles published between May 2022 and December 2023, allowing us to understand better the research being undertaken during the present outbreak. Compared to the previous bibliometric study, the extracted articles had no language constraints, providing a more comprehensive picture of the current literature.

The current bibliometric analysis has certain limitations. Most of our limitations align with previously published bibliometric analysis [25-27]. VOSviewer cluster maps were constructed without filtering articles by titles or abstract screening, which may result in inaccuracies in the keyword and author collaboration results. A probable length time-effect bias, like in previous bibliometric research, disadvantages more recent works in gaining citations. Furthermore, due to language limitations, non-English language articles may be misclassified.

## Conclusions

By analyzing papers from May 2022 to December 2023, the current study offered an overview of available research on the monkeypox virus during the current monkeypox virus outbreak. Our analysis identified the most influential research networks as being countries, institutions, journals, original articles, and authors. According to our findings, there is a growing demand for original research and for funding bodies to provide grants to expand existing research on the monkeypox virus. It can also be used to determine presentation patterns and the impact of monkeypox virus research. The current study can be utilized to better evaluate research needs and further the monkeypox virus research agenda.
